# Supplementary material for: Radiomics machine learning based on asymmetrically prominent cortical and deep medullary veins combined with clinical features to predict prognosis in acute ischemic stroke: a retrospective study
Source: PeerJ. 2025 Jun 6;13:e19469. doi: 10.7717/peerj.19469 (PMC12147764; doi:10.7717/peerj.19469)
Supplement: Supplemental Information 1 [file peerj-13-19469-s001.docx]

**Supplementary table S1:**

**MR scanning parameters for each sequence.**

|  | TR (ms) | TE (ms) | FOV (mm) | Slice thickness (mm) | matrices |
| --- | --- | --- | --- | --- | --- |
| T1WI | 1750 | 25 | 240x240 | 5 | 320x256 |
| T2WI | 4969 | 92.7 | 240x240 | 5 | 512x512 |
| FLAIR | 8400 | 145 | 240x240 | 5 | 256x256 |
| DWI | 4000 | 65.4 | 240x240 | 5 | 256x256 |
| SWI | 44 | 25 | 240x240 | 1 | 384x320 |

T1WI, T1-weighted imaging; T2WI, T2-weighted imaging; FLAIR, fluid attenuated inversion recovery; DWI, diffusion weighted imaging; TR, repetition time; TE, echo time; FOV, field of view.

**Supplementary table S2:**

**Comparison between different machine learning algorithms.**

| gorithm | DMV-APCV test set | | | | |
| --- | --- | --- | --- | --- | --- |
|  | Acc (95%CI) | Sen | Spe | PPV | NPV |
| ExtraTrees | 0.930 (0.8905 - 1.0164) | 0.952 | 0.909 | 0.909 | 0.952 |
| Random Forest | 0.930 (0.8541 - 1.0064) | 0.980 | 1.000 | 1.000 | 0.981 |
| DecisionTree | 0.512 (0.7569 - 0.9640) | 0.000 | 1.000 | 0.000 | 0.512 |
| SVM | 0.953 (0.8905 - 1.0164) | 0.952 | 0.955 | 0.952 | 0.955 |

DMV, deep medial vein; APCV, asymmetrically prominent cortical vein; SVM, support vector machine; Acc, Accuracy;

Sen, sensitivity; Spe, specificity; PPV, positive predictive value; NPV, negative predictive value.

In this study, We compared Random Forest (RF), ExtraTrees, DecisionTree and SVM algorithms. The results show that SVM has the best accuracy, sensitivity and specificity, and the joint model is optimal when SVM is combined with clinical indicators.

**Supplementary table S3:**

**DeLong test for the clinical model, APVC-DMV radiomic model and combined model in predicting the outcomes of AIS patients.**

|  | Combined vs Clinical | Combined vs APVC-DMV | APVC-DMV vs Clinical | Set |
| --- | --- | --- | --- | --- |
| P value | <0.001 | 0.038 | <0.001 | Training |
| P value | 0.009 | 0.039 | 0.038 | Test |

DMV, deep medial vein; APCV, asymmetrically prominent cortical vein; AIS, acute ischemic stroke.
